# Multiple variants of the human presequence translocase motor subunit Magmas govern the mitochondrial import

**DOI:** 10.1016/j.jbc.2021.101349

**Published:** 2021-10-29

**Authors:** Tejashree Pradip Waingankar, Patrick D'Silva

**Affiliations:** Department of Biochemistry, Indian Institute of Science, Bangalore, India

**Keywords:** mitochondria, mitochondrial translocase of inner membrane, protein import, protein translocation, Magmas, Co-IP, coimmunoprecipitation, DHFR, dihydrofolate reductase, GM-CSF, granulocyte-macrophage colony-stimulating factor, IM, inner membrane, IMS, intermembrane space, Magmas, mitochondria-associated granulocyte-macrophage colony-stimulating factor signaling molecule, mtHsp70, mitochondrial heat shock protein 70, MTS, mitochondrial targeting sequence, Ni-NTA, nickel-nitrilotriacetic acid, ROS, reactive oxygen species, TOM, translocase of outer membrane, TIM, translocase of inner membrane

## Abstract

Mitochondrial protein translocation is an intricately regulated process that requires dedicated translocases at the outer and inner membranes. The presequence translocase complex, translocase of the inner membrane 23, facilitates most of the import of preproteins containing presequences into the mitochondria, and its primary structural organization is highly conserved. As part of the translocase motor, two J-proteins, DnaJC15 and DnaJC19, are recruited to form two independent translocation machineries (translocase A and translocase B, respectively). On the other hand, the J-like protein subunit of translocase of the inner membrane 23, Mitochondria-associated granulocyte-macrophage colony-stimulating factor signaling molecule (Magmas) (orthologous to the yeast subunit Pam16), can regulate human import-motor activity by forming a heterodimer with DnaJC19 and DnaJC15. However, the precise coordinated regulation of two human import motors by a single Magmas protein is poorly understood. Here, we report two additional Magmas variants (Magmas-1 and Magmas-2) constitutively expressed in the mammalian system. Both the Magmas variants are functional orthologs of Pam16 with an evolutionarily conserved J-like domain critical for cell survival. Moreover, the Magmas variants are peripherally associated with the inner membrane as part of the human import motor for translocation. Our results demonstrate that Magmas-1 is predominantly recruited to translocase B, whereas Magmas-2 is majorly associated with translocase A. Strikingly, both the variants exhibit differential J-protein inhibitory activity in modulating import motor, thereby regulating overall translocase function. Based on our findings, we hypothesize that additional Magmas variants are of evolutionary significance in humans to maximize protein import in familial-linked pathological conditions.

In eukaryotic organisms, the organellar compartmentalization of cellular processes ensures metabolic homeostasis. Mitochondria are indispensable organelles vital for several cellular functions, including oxidative phosphorylation, β oxidation, iron-sulfur cluster biogenesis, and cell death (1, 2, 3, 4). The mitochondrial genome encodes only 8 and 13 proteins in yeast and humans, respectively. Hence, most mitochondrial protein repertoire is synthesized on the cytoplasmic ribosomes and subsequently imported into the organelle by organized protein-import machinery (5).

The mitochondrial precursor proteins with targeting sequence (MTS) are recognized by the translocase of the outer membrane (TOM) and inner membrane (TIM). TIM23 (also known as presequence translocase) is a dynamic complex owing to the import of precursor proteins into the matrix, inner membrane (IM), and intermembrane space (IMS) (6, 7, 8). The basic architecture of the TIM23 complex has remained conserved from yeast to humans. In *Saccharomyces cerevisiae*, there are two separate complex assemblies required for the matrix and IM sorting. The lateral IM sorting is performed by the core components of the TIM23 complex together with Tim21 and Mgr2 subunits (9, 10). The matrix import is aided by the coordination of the core channel and import-motor subunit (11). The core channel consists of the integral membrane proteins, Tim50, Tim23, and Tim17. The membrane receptor protein Tim50 is required for the presorting of incoming polypeptides from the TOM complex. The Tim23 forms a central twin pore that allows the nascent polypeptide to cross the IM, whereas Tim17 maintains the structural integrity and gating behavior of the channel (12, 13, 14, 15). The initial movement of the precursor proteins into the matrix is driven by the electrical membrane potential across IM followed by the active functioning of the import motor for complete translocation. The energy-dependent import is performed by the import-motor complex, whose subunits are highly conserved across species (16). Mitochondrial heat shock protein 70 (mtHsp70, Ssc1 in yeast) forms the core of the import motor tethered to the TIM23 channel *via* peripherally associated protein, Tim44 (17). The Ssc1 interacts with the short hydrophobic segments of the incoming polypeptide in an ATP-bound form. Its activity is stimulated by Pam18, a J-protein, thereby assisting the vectorial movement of the polypeptide chain to the matrix (18, 19, 20). The J-domain of Pam18 interacts with the J-like domain protein, Pam16, which regulates the import-motor activity, thereby maintaining the efficiency of the import (21, 22, 23). The Mge1 exchanges the nucleotide-bound form of Ssc1 (ADP to ATP), thus priming the further cycles of the import-motor activity (24).

Although the primary organization of the TIM23 complex was believed to remain conserved during evolution, there are significant differences due to multiple paralogs and limited understanding of the human mitochondrial import system. The structural annotations of the TIM23 subunits are well studied in yeast with the presence of a single presequence translocase channel. However, humans evolved to have multiple distinct TIM23 complexes. This is primarily because of the occurrence of additional variants of components of the TIM23 complex. For example, Tim17 exists in three forms, namely, Tim17a, Tim17b1, and Tim17b2, as subunits of separate TIM23 complexes (25, 26). Similarly, the nucleotide exchange factors such as Mge1 of yeast exist in two forms in humans with distinct functions, namely, GrpEL1 and GrpEL2 (27, 28). On the other hand, humans were evolved to have two J-proteins, DnaJC19 and DnaJC15, instead of single Pam18 in yeast (29, 30). Since J- and J-like proteins operate as a heterodimer, the presence of two J-proteins in humans raises the question about their regulation by a single form of the human counterpart of *S. cerevisiae* J-like protein, Magmas (Human ortholog of Pam16).

Mitochondria-associated granulocyte-macrophage colony-stimulating factor (GM-CSF) signaling molecule (Magmas) was first identified as a protein overexpressed upon GM-CSF signaling. The reduced expression of Magmas severely impaired the PGMD1 cell proliferation when grown in the presence of GM-CSF (31). The protein levels of Magmas were significantly elevated during the developmental stages in the heart, liver, muscles, pancreas, and testes (32). Later, a critical analysis highlighted the Magmas as a functional ortholog of yeast Pam16, a subunit of human import motor (33, 34). Structurally, the N-terminus of Magmas contains predicted noncleavable MTS, whereas the J-like domain resides toward the C-terminus. Magmas belong to the type IV class of J-proteins wherein the conserved HPD motif at the C-terminus is replaced by Aspartate-Lysine-Serine (35). Therefore, Magmas cannot stimulate the ATP hydrolysis activity of mtHsp70 (mortalin in humans) and functions as a subcomplex with J-proteins, DnaJC19 and DnaJC15, by regulating the import-motor activity at the presequence translocase machinery (36, 37). The Magmas function is essential for the development of *C. elegance*, and its homozygous deletion was lethal in *Drosophila* larva (38, 39).

Apart from the primary constitutive protein import role, Magmas was shown to have additional moonlighting functions in overexpressed conditions, presumably as a signaling molecule. The overexpression of Magmas is primarily detected in multiple cancer subtypes, including prostate, ovarian cancer, and malignant gliomas (40, 41, 42). Moreover, Magmas was potentially involved in the neoplastic transformation when overexpressed in ACTH-secreting pituitary adenomas and protected the cells from apoptosis (43, 44). In human cells, the Magmas overexpression suppresses oxidative stress by regulating the reactive oxygen species (ROS) levels (45). In a similar line, Magmas ortholog, black-pearl inhibition in *Drosophila melanogaster* resulted in increased ROS and decreased cell proliferation (46). Recently, in *Arabidopsis thaliana*, two paralogs of Pam16 were identified, namely, AtPAM16 and AtPAM16L, that are required for the mitochondrial import and plant immunity (47, 48). Hence, having the additional paralogs with evolved secondary roles raises an intriguing question about regulating Magmas levels in the mammalian system and its possible existence in multiple forms with different secondary moonlighting functions in pathological conditions.

In the present study, we first-time highlight the existence of two additional Magmas variants in humans. Both the variants of Magmas are recruited to the separate TIM23 machinery regulating import-motor functions, further supporting the presence of multiple translocation machinery at the mitochondrial IM. Our biochemical analyses provide compelling evidence to show that Magmas variants differentially regulate the import-motor activity, thus highlighting the physiological significance of the existence of the multiple forms in normal and pathological conditions.

## Results

### Identification of novel human Magmas variants

**Figure 1:**
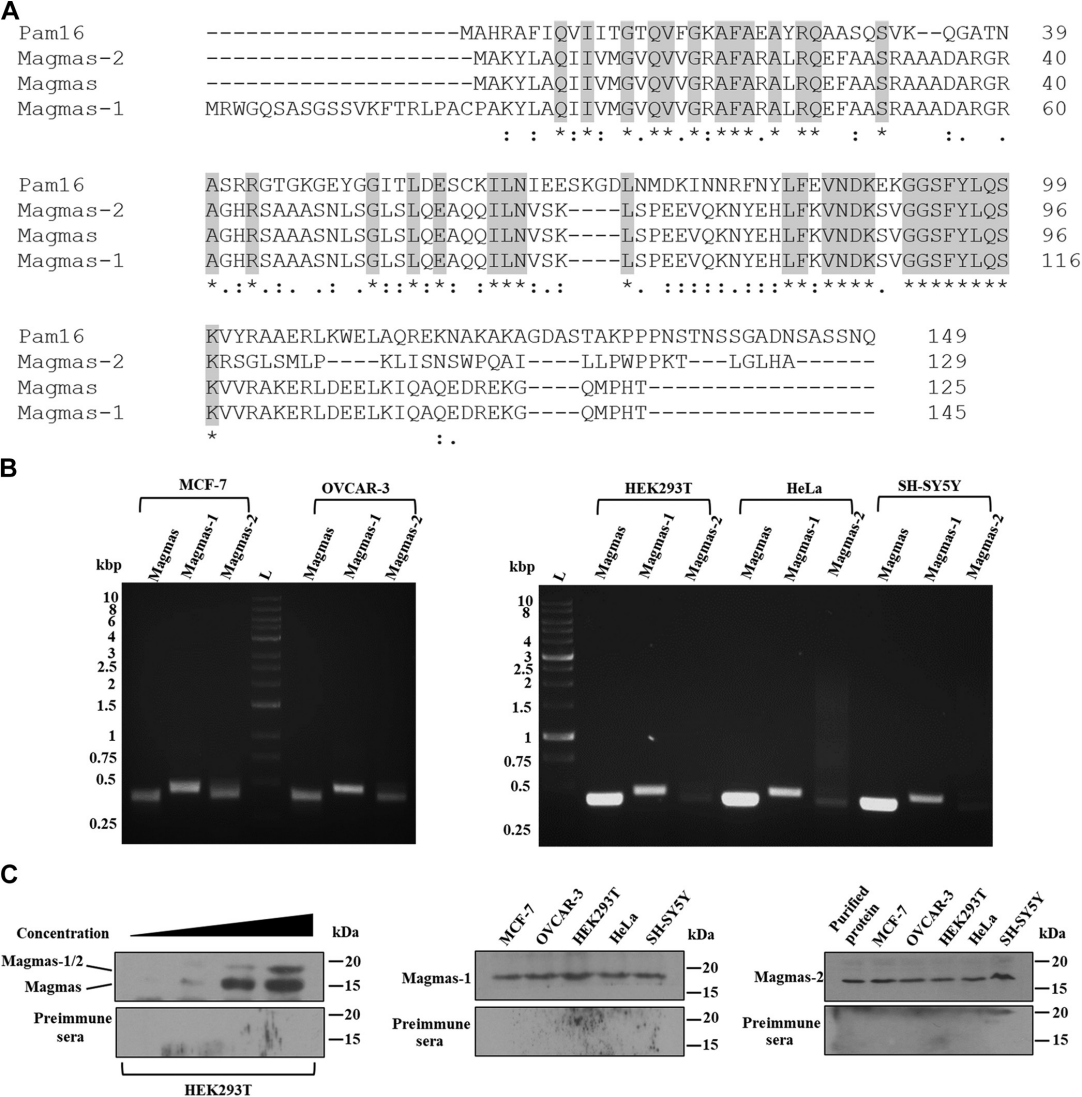
**Sequence analysis and relative expression of Magmas variants.***A*, depiction of Magmas variants amino acid sequence. Multiple sequence alignment of Magmas variants was performed using the Clustal Omega analysis tool compared with the yeast Pam16 sequence. The *gray color* marks identical residues. *B*, the expression of Magmas variants at the transcript level. cDNA isolated from MCF-7, OVCAR-3, HEK293T, HeLa, and SH-SY5Y cell lines were subjected to PCR analysis with the primers specific against the unique regions of Magmas variants. *C*, protein expression analysis of Magmas variants. Mitochondrial extracts isolated from HEK293T cells (to detect all the variants with a common J-like domain-specific antibody) were subjected to SDS-PAGE analysis in an increasing concentration-dependent manner, followed by immunodetection using an anti-Magmas antibody. Compared with Magmas (*lower band*), the Magmas-1 and Magmas-2 showed similar mobility on SDS-PAGE (*upper band*; highlighted). To resolve further, Magmas-1 and Magmas-2, the mitochondrial extract was isolated from indicated cell lines (MCF-7, OVCAR-3, HEK293T, HeLa, and SH-SY5Y) and separated on SDS-PAGE, followed by immunodecoration with specifically indicated antibodies (Magmas-1 or Magmas-2). Magmas, Mitochondria-associated granulocyte-macrophage colony-stimulating factor signaling molecule.

The TIM23 complex is an essential IM translocase for the importing and sorting of mitochondrial-targeted proteins. It forms a sole gateway for the biogenesis of matrix proteins aided by the membrane potential and import motor. Even though the basic architecture of the TIM23 complex has remained conserved during the evolution, the organization of the mammalian counterpart is far more intricate. The human TIM23 complex is described by the presence of multiple variant subunits performing distinguished functions. One of such protein paralogs identified in humans is the J-protein, DnaJC19 and DnaJC15. Both the J-proteins form a subcomplex with the Magmas and are organized into separate translocation machinery. Although two J-protein paralogs exist, how a single Magmas protein stoichiometrically regulates is still an open question. To test the presence of additional potential Magmas variants involved in regulating the protein import process in humans, we performed multiple sequence alignment with the Magmas sequence. Among the prey, two sequences with the highest homology were retrieved. One of the sequences (named hereafter as Magmas-1) had a unique extension at N-terminus, whereas the other evolved with a distinct C-terminus sequence (named hereafter as Magmas-2) (Fig. 1*A*).

To determine the existence of these variants at the cellular level, we examined their occurrence at both mRNA and protein expression levels. First, cDNA isolated from various human cell lines was analyzed by PCR with primers specific against the unique regions within the variant sequences. Interestingly, three amplicons were detected at an appropriate size, confirming the presence of multiple Magmas variants (Fig. 1*B*). Although the Uniprot database shows several predicted Magmas variant sequences (Uniprot entry number: A0A0A6YYL4, I3L167, I3L3T0, I3L1U7, I3L3G8, and A0A0B4J298), I3L3T0 and I3L1U7 were selected for this study and termed as Magmas-1 and Magmas-2, respectively. To rule out the presence of different variants, we performed multiple sequence alignment of the other predicted variants with Magmas, which resulted in very low sequence similarity. In addition, we designed the primers against the unique sequences in these predicted variants; however, PCR analysis of cDNA isolated from multiple human cell lines did not show amplification.

The steady-state protein expression was evaluated using antibodies explicitly recognizing the unique regions within the Magmas variants. First, the anti-Magmas antibody raised against the common J-like domain detected the presence of two Magmas variants (Fig. 1*C*, *panel 1*). However, because of the small difference in the molecular weights and similar mobility, we could observe only two forms of the variants in the SDS-PAGE (Fig. 1*C*, *panel 1*). The antibody specific for the J-domain of Magmas detected only two forms; therefore, we eliminated the other predicted variants of Magmas on the Uniprot database from the study. Secondly, we designed an antibody against the unique N- and C-terminus region of Magmas-1 and Magmas-2, respectively, which confirmed the existence of these variants in different cell lines (Fig. 1*C*, *panel 2* and *panel 3*). In summary, our results show the presence of two Magmas variants, Magmas-1 and Magmas-2, in multiple human cell lines that are probably formed as a result of alternate splicing events.

### Magmas variants functionally complement yeast Pam16 ortholog

**Figure 2:**
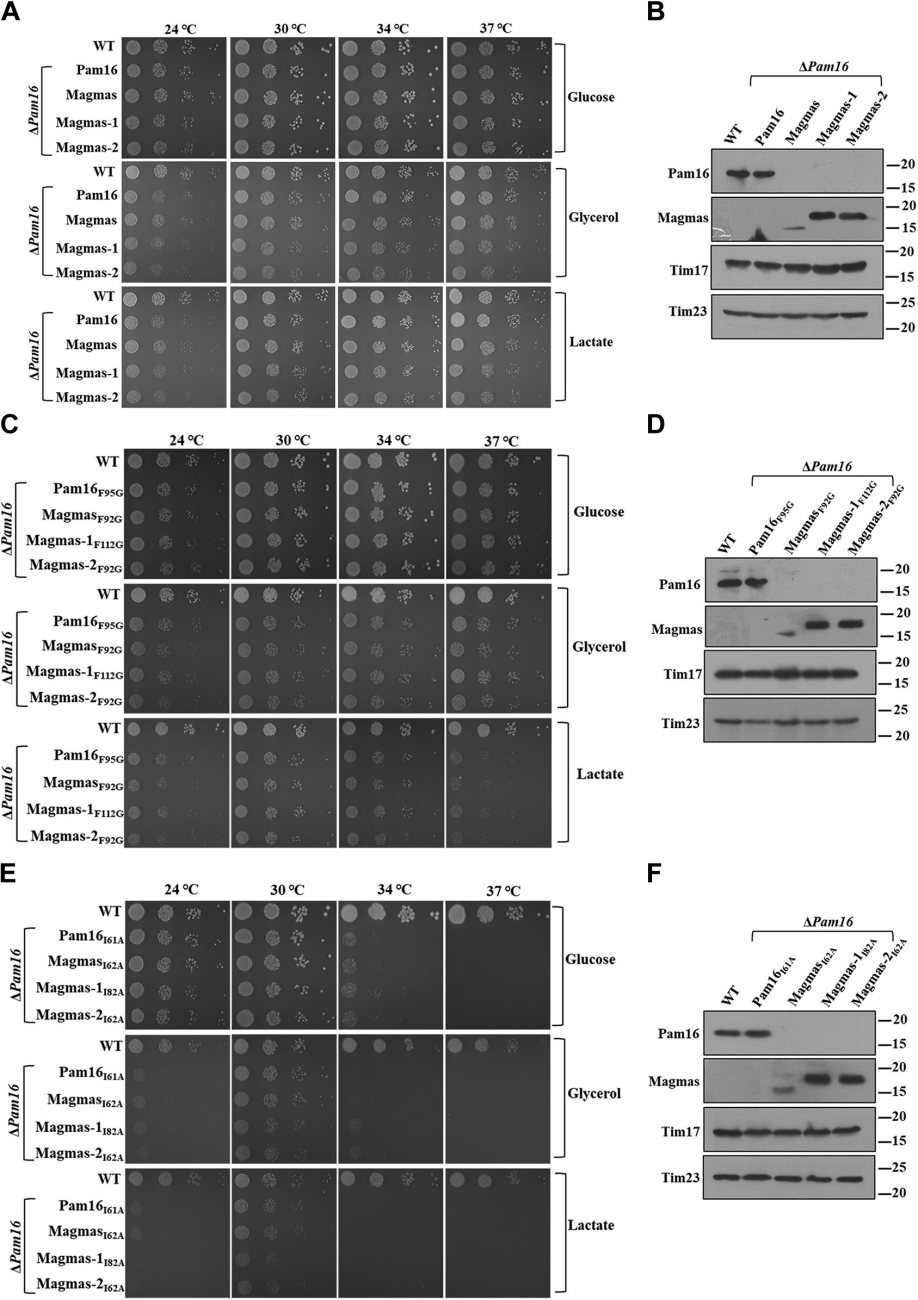
**Growth complementation analysis of Magmas variants.***A*, *C*, and *E*, growth phenotype analysis. An equal amount of yeast cells expressing WT and mutant Magmas variants were serially diluted, spotted on YPD, YPG, and YPL media, and incubated at indicated temperatures to assess *in vivo* growth complementation. *B*, *D*, and *F*, expression of steady-state protein levels of Pam16 and Magmas variants. The mitochondria isolated from cells expressing either Pam16 or Magmas were analyzed using antibodies specific for Pam16, Magmas, Tim23, and Tim17. Magmas, Mitochondria-associated granulocyte-macrophage colony-stimulating factor signaling molecule; TIM, translocase of the inner membrane.

The J-like protein, Pam16 of *S. cerevisiae*, has previously been reported as an ortholog of Magmas (33). Therefore, to functionally characterize the variants, Magmas-1 and Magmas-2 in yeast, we complemented the *S. cerevisiae* Δ*Pam16* strain. The growth complementation of Magmas variants was performed by the plasmid shuffling approach. The Magmas variants were expressed in pRS415_TEF_ yeast vector and transformed into Δ*Pam16* cells with Pam16 copy in a centromeric *URA3* based plasmid. The transformants were streaked on the 5-fluoroorotic acid, which selectively allowed the growth of cells devoid of *URA3*-Pam16 plasmid. The viable cells were recovered on minimal media and subjected to spot analysis on fermentable and nonfermentable carbon sources. As reported earlier, Magmas rescued the growth of Δ*Pam16* cells under all the conditions tested (Fig. 2*A*). Intriguingly, Magmas-1 and Magmas-2 also complemented yeast cell growth lacking Pam16 at all the temperatures on fermentable and nonfermentable carbon sources. The uncomplemented PJ53 background strain (WT) was used as a control, which exhibited comparable growth, suggesting that Magmas variants are equally functionally efficient (Fig. 2*A*). The expression of these variants is further validated by immunoblotting (Fig. 2*B*). The Magmas-1 and Magmas-2 were stably expressed, better than Magmas, exhibiting similar mobility in the SDS-PAGE. The steady-state expression of variants was higher even in the low expression centromeric yeast vector with ADH promotor, suggesting that unique N- and C-terminus extension sequences impart better stability or better translation rate with the variants in yeast (Fig. 2*B*).

Previous reports suggested that the helix-III region of Pam16 forms a functional heterodimer with Pam18 (36, 49). In addition, this region of Pam16 has remained conserved throughout the evolution, even in Magmas, suggesting its importance in maintaining mitochondrial homeostasis (33). Therefore, to determine the helix-III regions significance in the variants, we created a corresponding mutation reported earlier (F92G) and performed spot analysis on fermentative and nonfermentative carbon sources. *Magmas-1*_*F112G*_ and *Magmas-2*_*F92G*_ displayed a similar growth pattern to *Magmas*_*F92G*_ and *Pam16*_*F95G*_, suggesting functional conservation of this region in the variants (Fig. 2*C*). The expression of mutant proteins was validated by immunoblot analysis (Fig. 2*D*). Furthermore, to ascertain the role of the conserved J-like domain of Magmas, we generated a mutation outside the helix-III region of the J-like domain. Isoleucine at position 62 of Magmas was mutated to alanine in Magmas-1 and Magmas-2 to analyze the growth phenotype. *Pam16*_*I61A*_ and *Magmas*_*I62A*_ displayed a temperature-sensitive phenotype in glucose and glycerol media compared with WT, as reported earlier (33, 50). In addition, *Pam16*_*I61A*_ and *Magmas*_*I62A*_ showed severe growth defects at all the temperatures in lactate media compared with WT (Fig. 2*E*). Similarly, *Magmas-1*_*I82A*_ and *Magmas-2*_*I62A*_ had a similar growth pattern at all the temperatures studied on fermentable and nonfermentable carbon sources (Fig. 2*E*). The expression of mutants was confirmed by immunoblot analysis (Fig. 2*F*). Together, these results highlight the functional conservation of the J-like domain in Magmas variants, and residues in this region are critical for cellular growth.

### Magmas variants localize to the mitochondria and associate with the inner membrane

**Figure 3:**
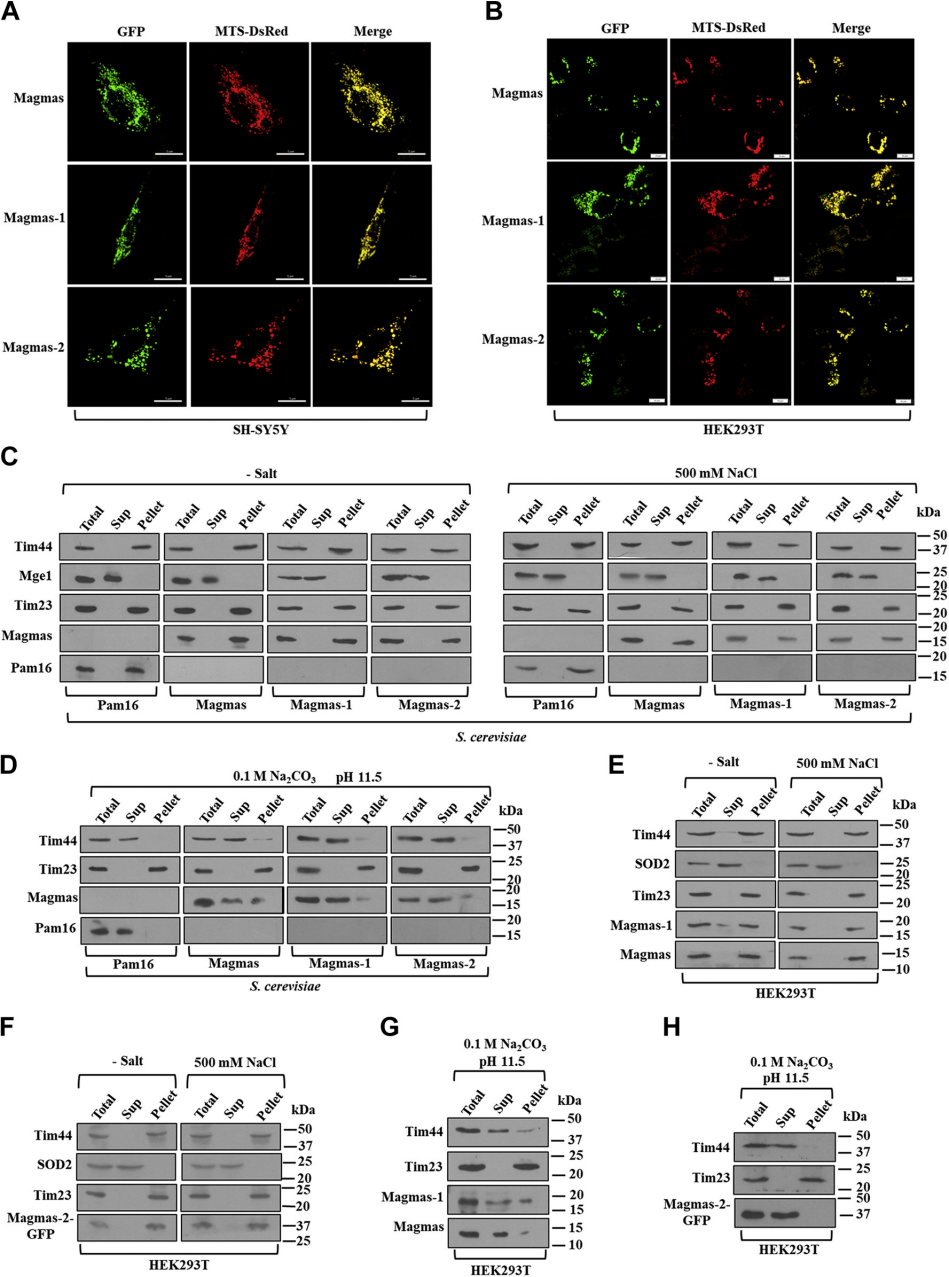
**Cellular localization of Magmas variants.***A* and *B*, intracellular localization of Magmas variants. GFP-tagged Magmas, Magmas-1, and Magmas-2, and MTS-DsRed were cotransfected into SH-SY5Y and HEK293T cells. The images were acquired after 48 h of transfection by Olympus FV3000. The scale bar for SH-SY5Y and HEK293T represents 5 μm and 10 μm. *C–H*, association of Magmas variants with the mitochondrial IM. The mitochondria isolated from yeast (*C*) and HEK293T (*E* and *F*) cells were subjected to hypotonic swelling followed by no salt or high salt (500 mM) treatment. The fractions (total; Supernatant [Sup]; pellet) were separated on SDS-PAGE and immunodecorated with the indicated antibodies. Similarly, the mitoplasts derived from yeast and human (*D*, *G–H*) mitochondria were subjected to carbonate extraction at pH 11.5. The fractions are separated by SDS-PAGE (total, supernatant (sup), and pellet), followed by immunodecoration using yeast and human-specific antibodies. IM, inner membrane; Magmas, Mitochondria-associated granulocyte-macrophage colony-stimulating factor signaling molecule; MTS, mitochondrial targetting sequence.

Magmas and Pam16 have been previously discovered to localize at the IM of mitochondria (21, 22). Therefore, we tested for the subcellular and subcompartment localization patterns of Magmas-1 and Magmas-2 by microscopy and fractionation analyses. To address this, we cloned Magmas variants at the N-terminus of GFP in the pEGFP-N3 vector and expressed them in SH-SY5Y and HEK293T cell lines (Fig. 3, *A* and *B*, *panel 1*). Mitochondria were decorated with constitutively expressing MTS-DsRed vector, which precisely targets DsRed to mitochondria because of MTS's presence (Fig. 3, *A* and *B*, *panel 2*). Magmas, Magmas-1, and Magmas-2 were found to exclusively colocalize with the DsRed with a resulting yellow fluorescence in the stained mitochondria (merge), suggesting mitochondrial targeting of the variants in the mammalian cells (Fig. 3, *A* and *B*, *panel 3*).

Although *in vivo* confocal imaging showed the mitochondrial distribution of variants, their precise destination within mitochondrial subcompartments remains elusive. To determine, we performed hypotonic swelling of mitochondria isolated from yeast and HEK293T cells to generate mitoplast. To investigate the IM localization, we resuspended mitoplast in high and low salt buffer followed by gentle sonication. Soluble supernatant and hydrophobic membrane pellet fractions were separated by ultracentrifugation and resolved on SDS-PAGE. The immunoblot analysis of Pam16 and Magmas was consistent with the previous findings (Fig. 3, *C*, *compare panels 1* and *2*). The variants, Magmas-1 and Magmas-2, were partitioned with the membrane pellet fraction similar to peripherally associated IM control protein, Tim44 (Fig. 3, *C,* *E,* and *F*, *compare panels 1–4*). As a control, Tim23, an IM spanning protein, was detected in the pellet fraction. On the other hand, matrix proteins SOD2 and Mge1 from human and yeast mitochondria, respectively, were used as controls for the soluble supernatant fractions (Fig. 3, *C,* *E*, and *F*, compare *panels 1–4*).

To distinguish whether the variants are associated peripherally or integrated into the IM, the mitoplasts derived from yeast and HEK293T mitochondria were subjected to alkaline extraction at pH 11.5. Both Pam16 and Magmas were extracted to the supernatant fraction consistent with the previously reported observations (Fig. 3, *D*, *G*, and *H*, *compare panels 1–2*). On the other hand, considerable amounts of Magmas-1 and Magmas-2, together with Tim44, were extracted in the supernatant fraction by alkaline pH treatment (Fig. 3, *G* and *H*, *compare panels 1–4*). The Tim23 protein was used as an integral membrane control that remained in the pellet fraction, suggesting that Magmas variants are peripherally associated with the IM of the mitochondria.

### Magmas variants associate with the presequence translocase machinery

The presequence translocase at the IM of mitochondria is responsible for importing matrix targeted proteins and IM sorting. It consists of membrane-embedded TIM23 core complex and dynamically peripherally associated import-motor subunits. Pam16 is shown to tether to the TIM23 complex *via* interacting through its J-like C-terminus domain by forming a heterodimer with Pam18, whereas the N-terminus interacts with Tim44 (23, 51). At the same time, Magmas interact with two J-protein partners (DnaJC19 and DnaJC15) to form separate subcomplexes at the human import motor, thereby organizing into multiple subtypes of the presequence translocase machinery (26, 37).

**Figure 4:**
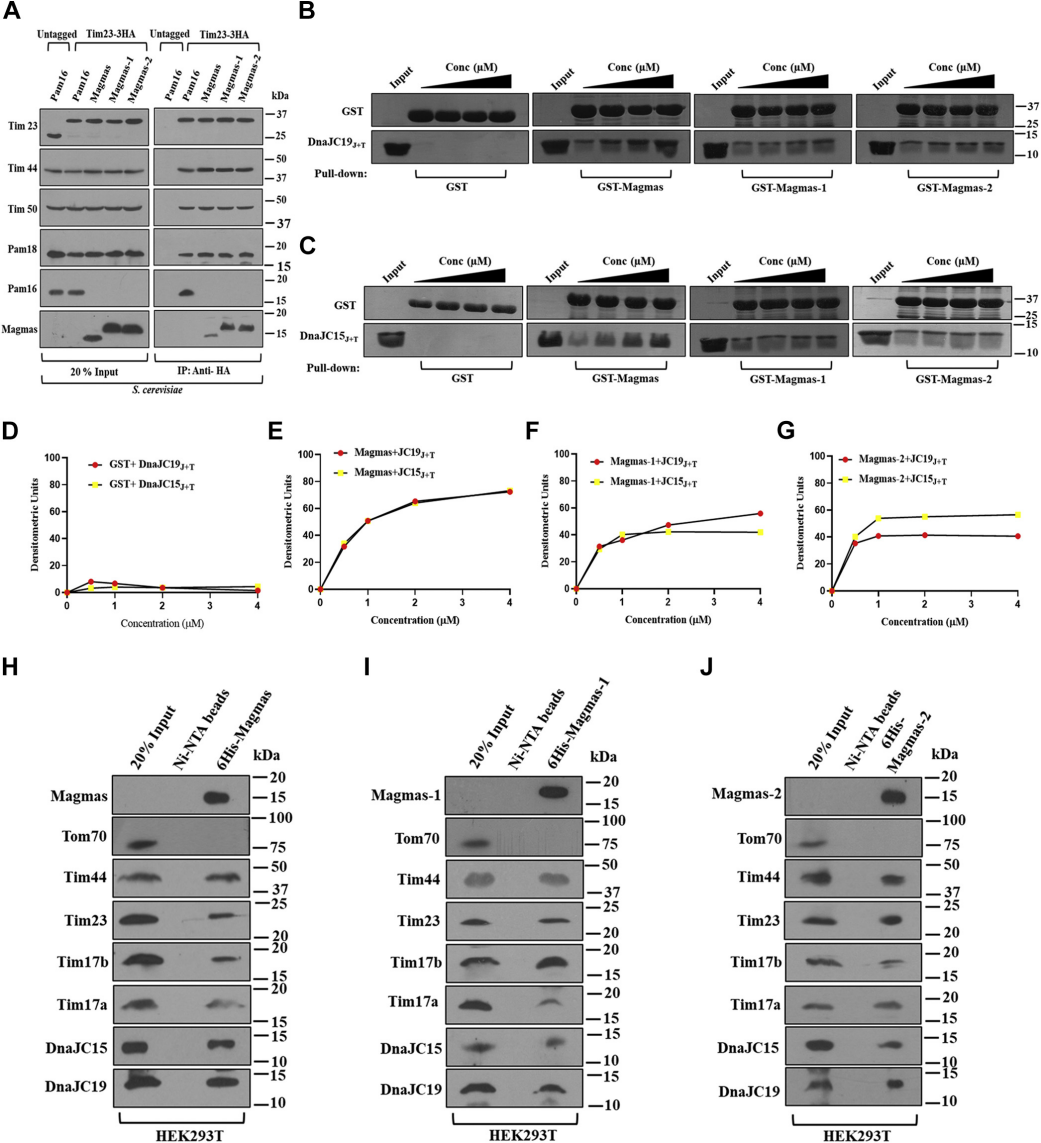
**Interaction analysis of Magmas variants with TIM23 complex.***A*, coimmunoprecipitation analysis of the yeast TIM23 complex. An equivalent amount of mitochondria isolated from HA-tagged and untagged Tim23 yeast strains were subjected to lysis using 1% digitonin. The soluble supernatant of mitochondrial lysate was incubated with the anti-HA conjugated protein G Sepharose beads. The samples were analyzed by immunodecoration using indicated antibodies. *B–G*, glutathione-S-transferase (GST) pull-down assay. 1 μM of GST alone or GST-Magmas variants were incubated with the increasing concentration of DnaJC19_J+T_ or DnaJC15_J+T_ at 24 °C for 45 min. The samples were separated by SDS-PAGE followed by Coomassie staining (*B* and *C*). The bands were quantified by densitometry by taking input as 100% and plotted against a function of concentration (*D–G*). GST alone was used as a negative control. *H–J*, Ni-NTA pull-down analysis of human TIM23 complex. Mitochondria isolated from HEK293T cells were lysed with the help of 0.5% NP40 followed by incubation with protein conjugated Ni-NTA beads. The pull-down samples were resolved by SDS-PAGE and detected using indicated antibodies. 20% of the lysate was used as an input loading control. Magmas, Mitochondria-associated granulocyte-macrophage colony-stimulating factor signaling molecule; Ni-NTA, nickel-nitrilotriacetic acid; TIM, translocase of the inner membrane.

To understand the association of Magmas variants with translocation machinery in both yeast and humans, we performed a coimmunoprecipitation (Co-IP) and pull-down analysis. Mitochondria were isolated from genomically tagged Tim23-3HA yeast strains expressing either Pam16, Magmas, Magmas-1, or Magmas-2 for Co-IP analysis. The mitochondrial lysate was prepared by nonionic detergent digitonin followed by the binding of supernatant with anti-HA antibody cross-linked protein G Sepharose beads. The Pam16 and Magmas were efficiently coprecipitated with TIM23 complex components such as Tim44, Tim50, and Pam18 consistent with earlier studies (Fig. 4*A*). On the other hand, Magmas-1 and Magmas-2 variants could coprecipitate with the TIM23 complex components, suggesting the functional subcomplex formation at the import motor (Fig. 4*A*). To validate the specificity of Co-IP, untagged Tim23 was used as a negative control, which did not show any coprecipitation of TIM23 subunits (Fig. 4*A*, *untagged, second panel*).

To evaluate the specificity of subcomplex formation between Magmas variants with human J-proteins, DnaJC19 and DnaJC15, we performed glutathione-S-transferase (GST) pull-down analysis. The *in vitro* binding was analyzed by incubating the equimolar amount of GST-Magmas variants with the increasing amounts of J-proteins. The Magmas showed equal affinity binding with both DnaJC19_J+T_ and DnaJC15_J+T_ (Fig. 4, *B*, *C*, and *E*). On the other hand, both the variants showed efficient binding with DnaJC19_J+T_ than DnaJC15_J+T_ (Fig. 4, *B*–*G*). However, Magmas-2 displayed better subcomplex formation with DnaJC15_J+T,_ albeit to a smaller extent than DnaJC19_J+T_ (Fig. 4, *B*, *C*, and *G*). The GST alone was used as a negative control, which did not show any pull-down of respective J-proteins (Fig. 4, *B*–*D*).

Although GST pull-down demonstrated competitive binding with J-proteins, the *in vivo* interaction of Magmas variants in the presence of other TIM23 complex components remains to be assessed. For analyzing the association with the human TIM23 complex, we performed a nickel-nitrilotriacetic acid (Ni-NTA) pull-down with hexahistidine tagged Magmas variants. Magmas was found to interact with TIM23 complex subunits, including Tim17a and Tim17b forms and the J-proteins DnaJC19 and DnaJC15 (Fig. 4*H*). Interestingly, on the other hand, Magmas-1 predominantly associated with the TIM23 complex containing Tim17b variant and DnaJC19, indicating that it majorly forms a part of essential presequence translocase B machinery (Fig. 4*I*). Intriguingly, the Magmas-2 was majorly found associated with the TIM23 complex containing the Tim17a and DnaJC15, thus predicting its predominant role in presequence translocase A machinery (Fig. 4*J*). Other components of the TIM23 complex, Tim23 and Tim44 were also detected in equal amounts in pull-down analyses validating the specific recruitment of Magmas variants to the presequence translocase machinery. The Ni-NTA beads alone and Tom70, a component of outer membrane TOM complex, served as a negative control, which did not show a significant pull-down of any components of the TIM23 complex (Fig. 4, *H*–*J*). In summary, our results highlight that the Magmas variants interact with the yeast presequence translocase and are differentially recruited to multiple human TIM23 types of machinery.

### Magmas variants perform import function at the mitochondrial inner membrane

**Figure 5:**
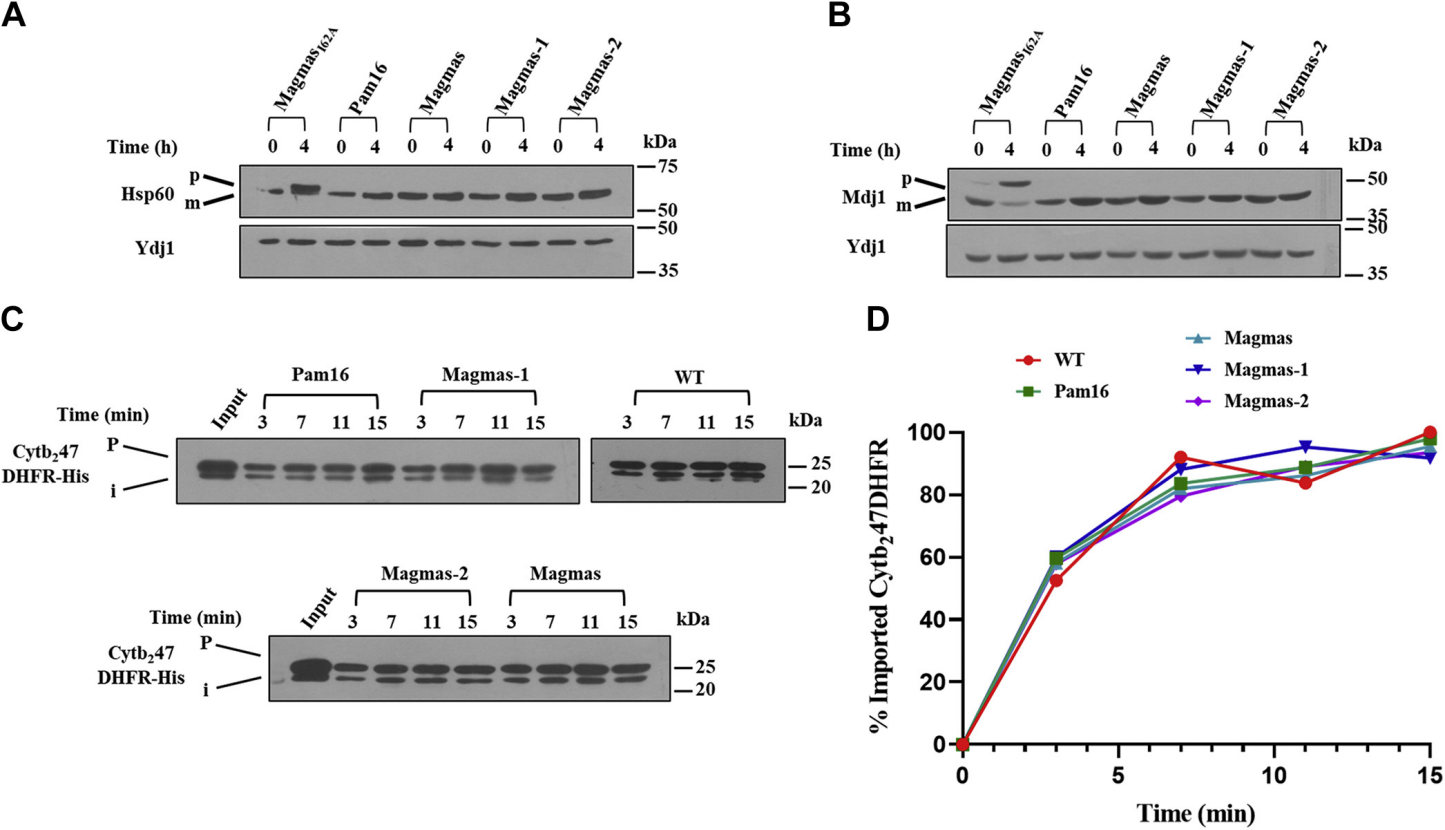
**Role of Magmas variants in import motor function.***A* and *B*, *in vivo* precursor accumulation assay. Yeast strains expressing Pam16, Magmas, Magmas-1, and Magmas-2 were grown at permissive temperature till the mid-log phase. Later, the cultures were shifted at 37 °C for 4 h to induce phenotype. The cell lysate was prepared from the samples to detect precursor protein accumulation using anti-Hsp60 and anti-Mdj1 antibodies. The temperature-sensitive mutant of Magmas (*Magmas*_*I62A*_) was used as an experimental control (33). p-precursor, m-mature (*C* and *D*) *in vitro* kinetic measurements of the import. The isolated yeast mitochondria from the strains mentioned above were incubated with Cytb_2_47DHFR, and import reaction was performed at indicated time points. The reaction was stopped by adding Valinomycin, followed by separation using SDS-PAGE and detection by anti-6His antibody. The protein imported at the last time point of WT was considered 100% to calculate the import rate. p-precursor, i-intermediate. Magmas, Mitochondria-associated granulocyte-macrophage colony-stimulating factor signaling molecule.

Pam16 and Magmas perform essential functions as a part of the import motor of the TIM23 complex. The matrix-targeted precursors are translocated *via* import motor, where J-like proteins (Pam16/Magmas) regulate J-protein activity, thereby assisting the precursor movement. Any defects in the import motor impair the mitochondrial matrix import leading to the accumulation of nuclear-encoded precursor proteins in the cytoplasm. The Magmas did not show any mitochondrial matrix protein import defects in Δ*Pam16* cells consistent with its ability to complement the growth. At the same time, we performed a precursor accumulation assay with Magmas-1 and Magmas-2 variants. We use two abundant yeast mitochondrial matrix proteins, Hsp60 and Mdj1, to determine *in vivo* precursor accumulation (52). Yeast cells (Δ*Pam16*) complemented with either Pam16 or Magmas variants were grown till the mid-log phase and subjected to heat shock. The cell lysate was resolved on SDS-PAGE followed by immunodecoration with anti-Hsp60 and anti-Mdj1 antibodies. The Magmas variants exhibited a negligible amount of unprocessed Hsp60 and Mdj1 precursors (Fig. 5, *A* and *B*). On the other hand, the Magmas temperature-sensitive mutant, *M*agmas_*I62A*,_ was used as an experimental control to demonstrate the precursor accumulation, exhibited an import defect (Fig. 5, *A* and *B*, *first two lanes*). The above results confirm that the Magmas variants can efficiently perform import functions as a part of the subunit of the TIM23 complex by complementing Pam16.

For the kinetic measurements of protein translocation into the mitochondrial matrix, we performed *in vitro* import assay with recombinant fusion protein Cytb_2_47dihydrofolate reductase (DHFR), as described previously (53). The mitochondria isolated from WT, Pam16, Magmas, and its variants expressing yeast strains were incubated with the saturated amount of substrate protein for the import reaction. Both Magmas-1 and Magmas-2 did not exhibit any defects in the import kinetics compared to WT, and Pam16 or Magmas, indicating that Magmas variants efficiently function at the yeast import motor (Fig. 5, *C* and *D*).

### Magmas variants differentially regulate the import-motor activity

**Figure 6:**
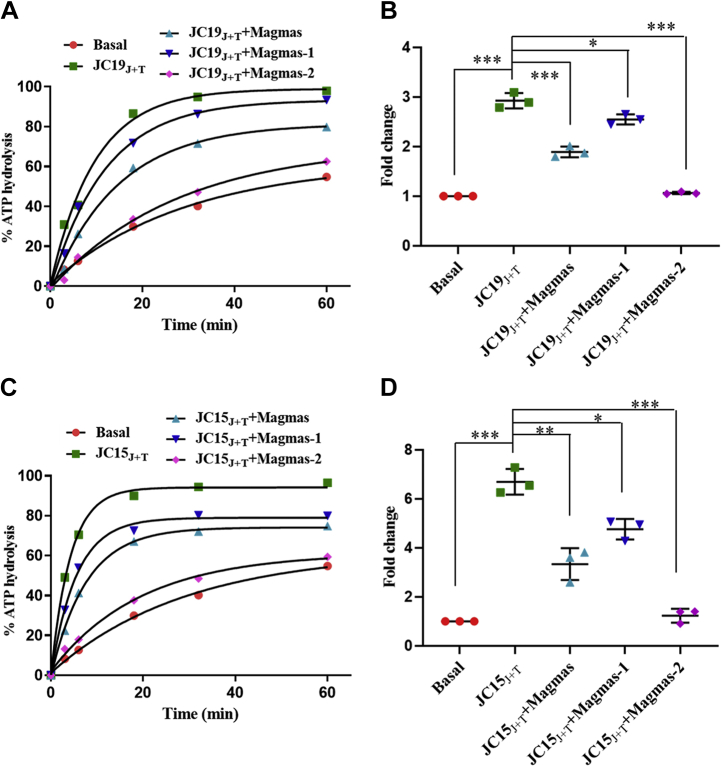
**Regulation of Mortalin ATPase activity by Magmas variants.***A–D*, The isolated purified proteins were subjected to ATP stimulation analysis with a 1:2:4 ratio of Mortalin-ATP (γ-^32^P): DnaJC19_J+T_(*A* and *B*)/DnaJC15_J+T_(*C* and *D*): Magmas variants. The ATP hydrolysis was monitored at the indicated time points. Relative fold change was calculated by considering the basal hydrolysis rate as 1. The normalized values are represented as a scatter plot. Statistical analysis was performed for three independent biological replicates (n = 3) using one-way ANOVA with Dunnett's multiple comparison test comparing all the columns with respective J-protein (∗*p* < 0.05, ∗∗*p* < 0.01, ∗∗∗*p* < 0.001). Magmas, Mitochondria-associated granulocyte-macrophage colony-stimulating factor signaling molecule.

The mtHsp70 is a weak ATPase and hence requires the J-protein partner(s) to stimulate the activity and drive the import reaction. Previously, DnaJC19 and DnaJC15 were shown to stimulate the ATP hydrolysis of mortalin at the human presequence translocase B and A, respectively (37). Further, Magmas downregulated this ATPase activity, thereby regulating the optimum import-motor function. Because Magmas variants associate with the human TIM23 complex, we tested their inhibitory action on the import motor. To assess, we performed the single turnover ATP stimulation assay. Mortalin alone showed a weak ATP hydrolysis activity, which was further stimulated by nearly three folds upon the addition of DnaJC19_J+T_ (Fig. 6, *A* and *B*). On the other hand, Magmas was able to inhibit around 50 to 60% of activity consistent with earlier reports (Fig. 6, *A* and *B*). However, at similar stoichiometric levels, Magmas variants exhibited differential inhibitory activities. The Magmas-1 variant showed reduced inhibitory activity (20–30%) compared with Magmas (Fig. 6, *A* and *B*). Intriguingly, on the other hand, Magmas-2 displayed robust inhibition (> 90%) compared with Magmas and Magmas-1 variant (Fig. 6, *A* and *B*). The differential inhibitory activities implicate that Magmas variants may have a unique role in regulating the import motor of the TIM23 complex.

Similar results were obtained in the case of DnaJC15_J+T_, where the addition of the J-protein led to a ∼7-fold increase in the ATP hydrolysis activity of mortalin (Fig. 6, *C* and *D*). Intriguingly, this stimulation was decreased to 50 to 60% by the addition of Magmas, whereas Magmas-1 had ∼20 to 30% inhibitory effect on the mortalin ATPase activity (Fig. 6, *C* and *D*). Remarkably, the addition of Magmas-2 significantly decreased the ATP hydrolysis (>90%), thereby confirming the robust inhibitory nature of this variant (Fig. 6, *C* and *D*). In summary, Magmas variants differentially modulate the ATPase activity of mortalin in the presence of J-protein, thus suggesting a distinguished evolved physiological role in regulating the import motor.

## Discussion

In eukaryotic cells, mitochondrial-protein trafficking is a highly regulated process involving intricate import channels of outer membrane and IM. One such complex machinery is the TIM23 complex, required for the quality control of IM-targeted and matrix-directed proteins. Our understanding of the presequence translocase-dependent import process is rudimentary mainly because the bulk of the information is derived from *S. cerevisiae* as a model system. On the other hand, in the mammalian system, several forms of the TIM23 complex exist at the IM because of the presence of multiple subunit paralogs, performing constitutive functions and responsible for the development of various pathological conditions.

**Figure 7:**
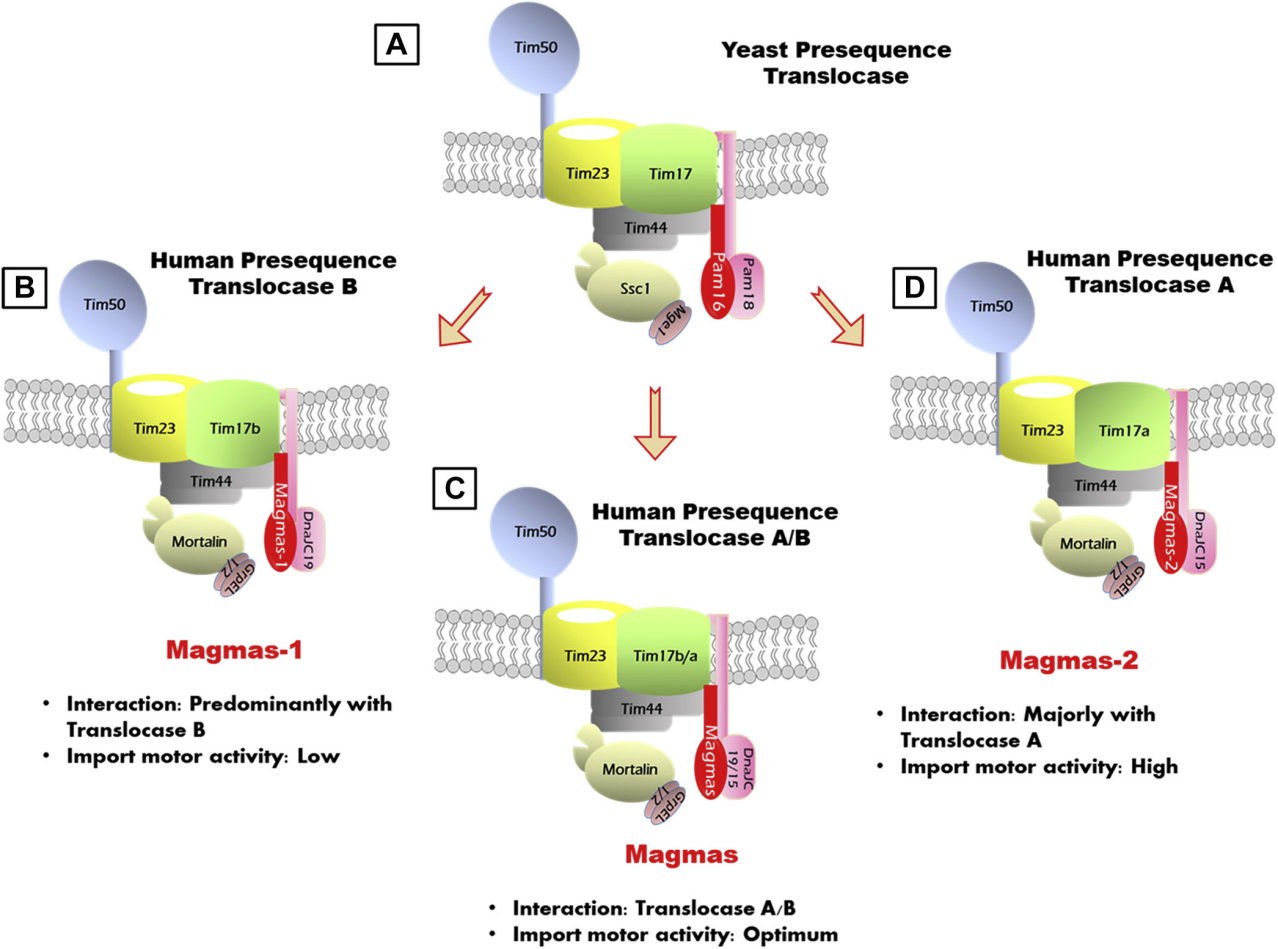
**Model depicting the evolution of Magmas at the presequence translocase from yeast to humans.***A*, a representation of yeast presequence translocase with single Pam16 forms the subcomplex with Pam18 of the import motor. *B*, the human presequence translocase machinery B (termed because of the presence of Tim17b paralog) with Magmas-1 predominantly forming subcomplex with DnaJC19 of the human import motor. *C*, the human presequence translocase machinery A/B (termed because of the presence of Tim17a/b paralog) with Magmas forming subcomplex with DnaJC15 and DnaJC19 at the human import motor. *D*, Magmas-2 is majorly associated with the translocase A by forming a subcomplex with DnaJC15. Magmas, Mitochondria-associated granulocyte-macrophage colony-stimulating factor signaling molecule; TIM, translocase of the inner membrane.

The present study highlights the first-time identification of two additional novel variants of Magmas that differentially regulate the human presequence translocase machinery. Our studies provide compelling evidence of the functional conservation of Magmas variants with *S. cerevisiae*, Pam16 ortholog. Though single Pam16 evolved from the lower eukaryotes to multiple variants in humans, the overall J-like domain structure remained functionally conserved, which was reflected by our mutational analysis in the yeast system. The Magmas variants are exclusively localized to mitochondrial IM by peripherally associating with the yeast TIM23 complex. This structural association of Magmas variants with yeast TIM23 complex resulted in the complete complementation of import-motor function as assessed by *in vivo* precursor accumulation and *in vitro* import kinetics. We propose the differentiation of TIM23 complexes based on Magmas variants interaction wherein each variant is predominantly associated with different parts of presequence translocase machinery (Fig. 7). Furthermore, the distinct functions of Magmas variants in regulating the import-motor activity highlight the significance of multiple forms. Previously, Magmas was reported to be involved in several disease manifestations; therefore, the presence of various Magmas variants with diverse activities may provide striking evidence of their biological significance in maintaining mitochondrial health.

Previous findings on the presequence translocase machinery supported an idea of the evolution of a single complex from yeast to multiple separate translocases in humans (namely translocase B and translocase A) (Fig. 7). Our findings further support this model in which, Magmas can interact with both translocase A and B (Fig. 7*C*). At the same time, Magmas-1 is predominantly associated with the components of translocase B machinery (Fig. 7*B*). On the other hand, Magmas-2 is majorly associated with translocase A (Fig. 7*D*). However, our pull-down analysis showed a small fraction of translocase A in Magmas-1 and translocase B in Magmas-2. Therefore, we hypothesize that the Magmas exist as a constitutively expressing primary form. At the same time, based on additional physiological demands in metabolically active tissues, Magmas-1 and Magmas-2 can further complement the import-motor subcomplex (Fig. 7, *B*–*D*). The physiological conditions under which this switch between Magmas variants occur remains an open question. Nonetheless, in humans, the presequence translocase machinery B is indispensable for normal cellular functioning, whereas translocase A has a designated oncogenic role (26). Based on our findings, we speculate that Magmas-1 may predominantly participate in a potential role in housekeeping functions, whereas Magmas-2 is probably majorly involved in pathological conditions such as oncogenesis.

At the TIM23 complex, the conserved J-like domain at the C-terminus of Magmas variants forms a heterocomplex with the J-domain of the J-protein, which essentially downregulates the ATPase activity of mortalin, thus regulating the import-motor function. In the case of Magmas-1, even after having a similar J-like domain to Magmas, it displayed lesser inhibition of ATPase activity with both the J-proteins. It is reasonable to believe that a combination of N-terminus extension and the J-like domain may impact the overall activity of the variant. Magmas-2 robustly inhibited the mortalin ATP hydrolysis, possibly because of the influence of the altered amino acid residues at the C-terminus on the J-like domain. Although many properties of the Magmas variants have remained evolutionarily conserved, we predict that these subtle differences at the N- and C-terminus may impact the overall functioning of the TIM23 complex in humans.

Understanding the secondary physiological role of Magmas and their variants in overall cell function and mitochondrial biogenesis is critical due to its differential expression in various metabolic tissues (32). The Magmas is overexpressed in cancer subtypes, including prostate, ovarian, and pituitary adenomas (40, 42, 43). These independent mitochondrial roles of Magmas, apart from the protein translocation, perhaps point toward its functioning as a signaling molecule under overexpressed conditions (41). Besides, Magmas demonstrated to have a novel moonlighting function as a ROS sensor by protecting cells against oxidative stress damage, and its ortholog is required for plant immunity (45, 47).

Recent genetic studies revealed that the homozygous mutations in a Magmas J-like domain with impaired mitochondrial import causes skeletal dysplasia in humans (54, 55). Although such missense mutations are deleterious in yeast, the severity of the phenotypes in humans raises intriguing questions. Pam16 and its orthologs such as Magmas are essential for cell viability. Under this homozygous mutational background in humans, it is possible that the Magmas-1 variant essentially can take over the Magmas constitutional functions as a part of the import motor at the presequence translocase, which is advantageous for the mammalian system. Similarly, a single point mutation in DnaJC19 (J-protein interacting partner of Magmas) resulted in the development of dilated cardiomyopathy with ataxia syndrome (29). Such mutations disrupt the subcomplex formation at the import motor, thereby impairing the import and mitochondrial biogenesis. So, having multiple variants of Magmas as a part of different translocase machinery may be highly beneficial to maximize the import efficiency, thus suppressing the adverse mutational phenotypic plasticity, which may be of evolutionary significance.

Besides, the DnaJC15 (second J-protein interacting partner of Magmas) levels were downregulated in response to chemotherapeutic reagents in cancer cells, thus imparting chemoresistance (56, 57). A small molecular inhibitor was recently designed to alter the Magmas functions in malignant gliomas as a therapeutic application (41). So, discovering new Magmas variants brings several important questions regarding their constitutive role in mitochondrial functions or how they serve as an effective additional therapeutic target. At the same time, there are other possible secondary functional attributes of variants to be explored in the future, specifically how they are involved in reprogramming the mitochondrial parameters in pathological conditions such as different cancer subtypes.

In conclusion, even though recent identifications confirmed the multiple TIM23 complexes in human mitochondria, its structural organization was enigmatic. Our identification of new Magmas variants further strengthens the idea of multiple presequence translocase machinery regulating the protein import across the human mitochondrial IM. Moreover, our biochemical analysis provides vital insight into understanding the human import motor assembly at the presequence translocase. So, identifying the new variants opens up new avenues to explore their participation in various pathological conditions.

## Experimental procedures

### Cells, cell culture, and yeast strains

HEK293T, HeLa, OVCAR-3, and MCF7 cells were cultured in Dulbecco's modified Eagle's medium (Invitrogen) supplemented with 10% fetal bovine serum ( Gibco), 1% Glutamax (Gibco), and 1% penicillin-streptomycin (Gibco). SH-SY5Y cells were grown in DMEM/F12 (Invitrogen) supplemented with the same components mentioned above. Transfection of HEK293T and SH-SY5Y was performed using Lipofectamine 2000 reagent (Invitrogen) as per the manufacturer instructions. For *in vivo* analysis in yeast, haploid W303 derivative, PJ53 background strain of *S cerevisiae* was used (WT) (Genotype: *trp1-1/trp1-1, ura3-1/ura3-1, leu2-3112/leu2-3/112, his3-11,15/his3-11,15 ade2-1/ade2-1, can1-100/can1-100 GAL2*^*+*^*/GAL2*^*+*^, *met2-Δ1/met2-Δ1, lys2-Δ2/lys2-Δ2*). The Magmas variants were transformed in Δ*pam16* strain carrying Pam16 in *URA3*-based plasmid and subsequently plated on minimal media. The transformants were streaked on 5-fluoroorotic acid plates to select the cells devoid of *URA3* plasmid. The resulting strains expressing the Magmas variants were used for spot analysis on YPD, YPG, and YPL media and other experiments.

### cDNA generation, primer designing, and cloning

The total RNA content was isolated with TRI reagent (Sigma) by following the manufacturer instructions. The RNA was used to generate cDNA using the iScript cDNA synthesis kit (Bio-Rad). The following primers were used for the detection of Magmas variants from cDNA: Magmas-F, 5′-ATGGCCAAGTACCTGGCCCAGAT C-3′, Magmas-R, 5′-CGTATGGGGCATC TGCCC-3′, Magmas-1-F, 5′-ATGAGGTG GGGCCAGTCTGCCTCTGG-3′, Magmas-1-R, 5′-CGTATGGGGCATCTG CCC-3′, Magmas-2-F, 5′-ATGGCCAAG TCCTGGCCCAGATC-3′, and Magmas-2-R, 5′-TCAGGCATGTAGTCCCAGCGTTTT GGGAGG-3′. The Magmas variants and Pam16 mutants were cloned in the pRS415 expression vector under the *TEF* promoter to complement the yeast strains. Full-length Magmas, Magmas-1, and Magmas-2 were purified as hexahistidine, or GST tagged protein by pET28 or pGEX-KG vectors, respectively. Localization studies were performed utilizing full-length sequences of the Magmas variants at the 5′ end of GFP in the pEGFP-N3 vector.

### Antibody generation

The antibody detecting Magmas-1 and Magmas-2 were raised against the N- and C-terminus unique peptide sequences, MRWGQSASGSSVKFTRLPACP and NSWPQAILLPWPPKTLGLHA, respectively (Abgenex). Human primary antibodies used in this study are previously described (26). Briefly, anti-Tim23 (BD Biosciences), anti-Tim44 (Sigma), anti-Tim17a (Epitomics), anti-Tim50 (Imgenex Biotech), anti-Magmas, anti-DnaJC19, and anti-DnaJC15 antibodies were raised against the respective J-like and J-domain, whereas anti-Tim17b antibody was generated against the C-terminus peptide CPKDGTPAPGYPSYQ (Imgenex Biotech). In addition, antibodies specific for yeast proteins, anti-Tim44, anti-Pam18, anti-Mge1, and anti-Pam16, were kindly gifted by Prof. Elizabeth A. Craig's laboratory. Anti-Tim23 and anti-Tim50 were constructed against the N-terminus (1–98) amino acids and C-terminus (133–476) amino acids, respectively.

### *In vivo* cell imaging and mitochondrial fractionation

SH-SY5Y and HEK293T cells were cotransfected with GFP-tagged Magmas variants fusion protein and MTS-DsRed constructs. After 48 h of transfection, the images were acquired using Olympus FlowView3000. Yeast and human mitochondria were isolated, as previously described (58, 59). 0.6 mg of yeast and 1 mg of human mitochondria were allowed to undergo hypotonic swelling in sonication buffer (20 mM Hepes-KOH pH 7.5, 150 mM KCl, 10 mM magnesium acetate, and 1 mM phenylmethylsulfonyl fluoride [PMSF]) for 10 min. For high salt conditions, 500 mM NaCl was added to the buffer before sonication, whereas for peripheral protein fractionation, 0.1 M Na_2_CO_3_ pH 11.5 was added after sonication. Mitoplast lysis was achieved by sonication later; the lysate obtained was subjected to ultracentrifugation at 50,000 rpm for 30 min at 4 °C (Optima TLX table-top ultracentrifuge [Beckman-Coulter]). The resulting supernatant and pellet fractions were further separated on SDS-PAGE, and the proteins of interest were detected by immunoblot analysis.

### Protein purification

Purification of hexahistidine tagged DnaJC19_J+T_ (Amino acids: 34–112) and DnaJC15_J+T_ (Amino acids: 69–146) was carried out, as described earlier (33, 37). The Magmas variants (full-length) were purified by standard affinity chromatography using Ni-NTA beads (GE Healthcare). Briefly, *Escherichia coli* (RIL) expression strain with the Magmas variants was allowed to grow till the mid-log phase. The culture was induced by the addition of 1 mM IPTG for 6 h at 30 °C. Cell pellet obtained was incubated in 20 ml of buffer A (20 mM HEPES-KOH pH 7.5, 20 mM Imidazole, 100 mM KCl, and 10% glycerol) containing 0.2 mg/ml lysozyme and 10 mM PMSF for 1 h at 4 °C. The sample was lysed with 0.2% deoxycholic acid for 15 min at 4 °C followed by sonication at 23% amplitude. The unlysed cells and debris were separated by centrifugation at 16,000 rpm for 45 min, and the clear soluble supernatant was incubated with Ni-NTA beads for 2 h at 4 °C. The unbound proteins were removed by washing twice with buffer A containing 0.01% TritonX-100 followed by a wash with buffer B (20 mM HEPES-KOH pH 7.5, 80 mM Imidazole, 100 mM KCl, and 10% glycerol). Bacterial DnaK contamination was removed by washing the resins with buffer C (20 mM HEPES-KOH pH 7.5, 50 mM Imidazole, 100 mM KCl, 0.1 mM ATP, 10 mM MgCl_2_, and 10% Glycerol). Finally, proteins were eluted in the elution buffer (20 mM HEPES-KOH pH 7.5, 100 mM KCl, 300 mM Imidazole, and 10% glycerol). For the purification of GST tagged proteins, *E. coli* (C41) expression strain was used. The proteins were isolated and purified according to the previously published protocol with a minor modification (60). Tris-HCl pH 7.5 was used instead of phosphate buffer in the purification process.

### Coimmunoprecipitation, Ni-NTA, and GST pull-down assay

For the interaction studies of the yeast TIM23 complex, 2 mg of mitochondria were subjected to lysis in Co-IP buffer [25 mM Tris-HCl (pH 7.5), 10% glycerol, 80 mM KCl, 5 mM EDTA, and 1 mM PMSF] containing 1% digitonin (Calbiochem). The lysates were then centrifuged at 14,000 rpm for 20 min at 4 °C to remove the unlysed mitochondria. The clear supernatant was incubated for 3 h with primary antibody-bound protein G Sepharose beads (GE Healthcare) at 4 °C. Subsequently, the samples were washed in a lysis buffer containing 0.5% digitonin followed by separation on SDS-PAGE and detected by immunoblot analysis with specific antibodies. For GST pull-down analysis, the equimolar concentration of GST tagged Magmas variants were incubated with buffer A (50 mM Tris-HCl pH 7.5, 100 mM KCl, 0.2% Triton-X 100, 10% Glycerol, and 1 mM PMSF) for 10 min at 24 °C followed by blocking with 0.1% bovine serum albumin for 20 min at 24 °C. GST alone or GST-Magmas variants were then incubated with increasing concentrations of either DnaJC19_J+T_ or DnaJC15_J+T_ for 45 min at 24 °C. The samples were washed twice with buffer A and subjected to SDS-PAGE analysis followed by Coomassie dye staining. 2 mg mitochondria, isolated from HEK293T cells, were incubated with lysis buffer (25 mM HEPES-KOH [pH 7.5], 80 mM KCl, 5 mM EDTA, 20 mM imidazole, 10% glycerol, and 1 mM PMSF) containing 0.5% NP-40 for 20 min under cold conditions. Clear supernatant obtained after centrifugation was employed for the pull-down assay with respective protein-bound beads for 12 h at 4 °C. The unbound or nonspecifically bound proteins were removed by washing with lysis buffer comprising 0.25% NP-40. The samples were resolved on SDS-PAGE and detected by immunoblot analysis using specific antibodies.

### Precursor accumulation assay

For the detection of precursor protein *in vivo*, the yeast cells expressing respective proteins were allowed to grow till the mid-log phase. The culture was then subjected to heat shock at 37 °C for 4 h. The cell lysate was prepared and separated using SDS-PAGE, followed by immunoblotting using anti-Hsp60 and anti-Mdj1 antibodies. *In vitro* import kinetics was performed, as described earlier (53). The substrate used in this assay was a matrix targeting Cytb_2_47DHFR. This substrate was made by fusing the nucleus-localized dihydrofolate reductase with the N-terminus MTS of cytochrome b_2_ (Cytb_2_). Saturating amount of substrate was incubated with the isolated energized mitochondria. The reaction was stopped by the addition of Valinomycin followed by Western blot analysis using anti-6His antibody.

### ATP stimulation assay

The J-proteins, DnaJC19 and DnaJC15, were isolated with the N-terminus truncation and essentially having J-domain and predicted MTS (J + T). A complex of Mortalin with γ-^32^P labeled ATP was incubated with DnaJC19_J+T_ or DnaJC15_J+T_ and the Magmas variants in the ratio of 1:2:4 at 25 °C. The reaction was stopped by adding stop solution (5 M LiAc, 17 M HCOOH, and 0.1 M ATP in the ratio 10:6:9) at indicated time points. Each reaction mixture was spotted on the PEI-cellulose plate and separated by thin-layer chromatography followed by acquiring images *via* Typhoon FLA 9000 (GE Healthcare).

## Data availability

All data produced in this study is provided in the manuscript.

## Conflict of interest

The authors declare that they have no conflicts of interest with the contents of this article.
